# Perception of the COVID-19 Pandemic Among Patients With Inflammatory Bowel Disease in the Time of Telemedicine: Cross-Sectional Questionnaire Study

**DOI:** 10.2196/19574

**Published:** 2020-11-02

**Authors:** Fabiana Zingone, Monica Siniscalchi, Edoardo Vincenzo Savarino, Brigida Barberio, Linda Cingolani, Renata D'Incà, Francesca Romana De Filippo, Silvia Camera, Carolina Ciacci

**Affiliations:** 1 Inflammatory Bowel Disease Center Department of Surgery, Oncology, and Gastroenterology University of Padua Padua Italy; 2 Inflammatory Bowel Disease Center Department of Medicine, Surgery, Dentistry, Scuola Medica Salernitana, University of Salerno University Hospital San Giovanni di Dio e Ruggi d’Aragona Salerno Italy

**Keywords:** COVID-19, IBD, ulcerative colitis, Crohn disease, telemedicine, biologic agents, perception, survey

## Abstract

**Background:**

After the COVID-19 outbreak, the Italian Government stopped most regular health care activity. As a result, patients with inflammatory bowel disease (IBD) had limited access to outpatient clinics and hospitals.

**Objective:**

This study aimed to analyze the perception of the COVID-19 emergency among patients with IBD during the early weeks of the lockdown.

**Methods:**

We invited adult patients with IBD from the University of Salerno (Campania, South Italy) and the University of Padua (Veneto, North Italy) by email to answer an ad hoc anonymous survey about COVID-19. We also collected data on demographic and disease characteristics.

**Results:**

In total, 167 patients with IBD from Padua and 83 patients from Salerno answered the survey (age: mean 39.7 years, SD 13.9 years; female: n=116, 46.4%). We found that patients with IBD were particularly worried about the COVID-19 pandemic (enough: 77/250, 30.8%; much/very much: 140/250, 56.0%), as they felt more vulnerable to COVID-19 due to their condition (enough: 70/250, 28.0%; much/very much: 109/250, 43.6%). Patients with IBD from the red zone of Veneto were more worried than patients from Campania (*P*=.001), and men felt more susceptible to the virus than women (*P*=.05). Additionally, remote medicine was appreciated more by younger patients than older patients (*P*=.04).

**Conclusions:**

The results of our survey demonstrate that the lockdown had a significant impact on the psychological aspects of patients with IBD and suggest the need for increasing communication with patients with IBD (eg, through telemedicine) to ensure patients receive adequate health care, correct information, and proper psychological support.

## Introduction

The COVID-19 pandemic is a public health emergency [1]. Studies from China, the country where the SARS-CoV-2 outbreak initially occurred, have indicated that the potential risk factors for poor prognoses in people who contract the SARS-CoV-2 virus include male gender, older age, high Sequential Organ Failure Assessment scores, and high D-dimer levels [2,3]. The extraordinary measures that have been implemented to contain viral spread, such as the cancellation of flights, the initial lockdown of large areas in China, and the subsequent lockdown of large areas in Korea and Italy, have captured the attention and interest of the public. However, these measures have also generated misconceptions and fear [4].

In Italy, the first person-to-person virus transmission was reported by authorities on February 21, 2020. One of the first places to experiment with quarantine and lockdown was Vo’, a small town near Padua in Veneto, on February 23, 2020. Subsequently, after a few days, lockdown measures were extended to the whole country. Outside of the lockdown measures adopted in China to combat the outbreak, the lockdown measures adopted in Italy were considered to be the most radical. Italians were not allowed to leave their houses. This excluded those who worked in essential services, such as health care, transportation, and supermarkets. Additionally, only one person per family was allowed to go out for food shopping. Further, while outside, the Italian population was asked to wear surgical masks and gloves. Some regions of Northern Italy, such as Veneto, were defined as red zones due to the high number of people infected with SARS-CoV-2 and deaths caused by SARS-CoV-2 infection. Moreover, the mass media and the internet have put significant pressure on the population due to the potentially lethal implications of SARS-CoV-2 infection. Therefore, due to the restrictions that have been implemented since the beginning of the quarantine, public and private health care workers have only been able to address emergencies.

Patients with inflammatory bowel disease (IBD) are at a higher risk of infection than the general population. This is mainly due to the immunosuppressive therapy that patients with IBD receive, which involves steroids, thiopurines, and biologic agents [5-8]. A large study demonstrated that patients with IBD had an increased risk of influenza infection and were more likely to require hospitalization than those without IBD [5]. However, the magnitude of this increased risk of infection and how it relates to the use of available medical therapies remains controversial [9]. Some studies have indicated the possibility of major life events inducing relapses in patients with IBD [10,11]. During the COVID-19 pandemic, patients with IBD have had to limit their access to outpatient clinics and hospitals. However, a vast majority of patients with IBD at our outpatient clinics have continued their immunosuppressant or biologic therapy. Health care providers have had to rapidly and abruptly convert their traditional methods for practicing medicine to the more modern concept of telemedicine [12]. For instance, phone calls or internet video calls via patient-available apps (eg, Skype, WhatsApp, Zoom) and emails have been used to contact patients with IBD and continue their follow-up. The aim of this study is to report the perception of the COVID-19 emergency among a cohort of patients with IBD who live in a red zone during the early weeks of lockdown and their ability to access remote consultations in order to improve their health care during possible future emergencies similar to the COVID-19 pandemic.

## Methods

During quarantine, personnel from IBD centers advised patients to postpone their regular outpatient visits if patients’ reasons for visiting were not urgent. They also invited patients to have remote health care consultations. With regard to regular pre-COVID-19 face-to-face outpatient visits in Italy, patients used to receive a report regarding drug prescriptions and lab tests, which patients were to return during the next follow-up visit. With telemedicine, patients in Italy are given their lab test results by email or fax prior to remote health care consultation. These results, along with patient evaluations for general health status, are then discussed during follow-up visits performed via remote consultation

**Figure 1:**
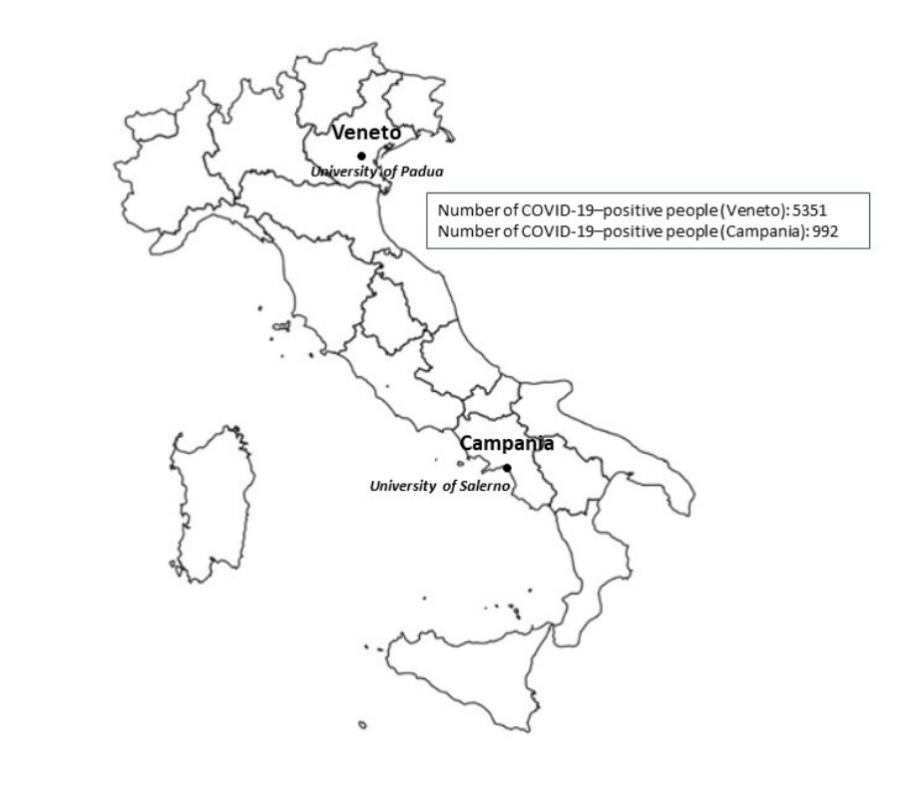
Map of Italy detailing the spread of COVID-19. The map was last updated on March 24, 2020 for Veneto and Campania.

Between March 14 and 24, 2020, we invited patients with IBD from the IBD Units of the University of Salerno (Campania region, South Italy) and the University of Padua (Veneto region, North Italy) (Figure 1) by email to answer an ad hoc anonymous survey about COVID-19. Similar surveys have been used at our centers for two other chronic conditions [13,14]. Patients provided their email address and consented to contact. Therefore, the survey used in this study follows the CHERRIES (Checklist for Reporting Results of Internet E-Surveys) checklist [15]. Patients enrolled from both universities only included adult patients with a confirmed IBD diagnosis and an equal distribution of men and women. The anonymous web survey included 14 multiple-choice questions that aimed to evaluate patients’ perception of the COVID-19 pandemic. In particular, we asked if patients were worried about the epidemic. The survey included questions about whether patients felt more susceptible to SARS-CoV-2 infection compared to the general population, whether they preferred to avoid going to the hospital, and their thoughts about remote consultation. We recorded patients’ sex, date of birth, time of IBD diagnosis, region of origin (Veneto or Campania), and type of disease (ie, Crohn disease, ulcerative colitis, and unclassified disease) [16]. We also asked for information regarding patients’ ongoing therapy and comorbidities. Patients also reported whether they had received the influenza vaccination. We expected an average time of 10 minutes to complete the survey. We sent a gentle reminder 5 days after the request was sent. We closed the survey to patients 10 days after it was sent out and analyzed the survey data.

Categorical and continuous variables were expressed as proportions with percentages and means with standard deviations, respectively. Comparisons between categorical variables were performed using the Chi-square test. A *P* value of <.05 was considered statistically significant. STATA 11 software was used for statistical analysis.

## Results

Of the 450 surveys sent, 250 (55.5%) were answered. In total, 167 patients from Veneto (age: mean 39.1 years, SD 13.5 years; male: n=91, 54.5%) and 83 patients from Campania (age: mean 39.1 years, SD 14.8 years; male: n=43, 51.8%) answered the survey. There were no statistically significant differences in age and sex between patients from Veneto and those from Campania. Of the 250 patients who answered the survey, 142 (56.8%) had Crohn disease and 100 (40.0%) had ulcerative colitis. The mean time from diagnosis to the point of the survey was 10.7 years (SD 10.6 years). More than half of our study population was on immunosuppressive/immunomodulatory therapy (138/250, 55.2%). Less than 15% (36/250) of patients had at least one other chronic disease, including diabetes, high blood pressure, and dyslipidemia (Table 1).

**Table 1:** Study population characteristics (N=250).

Characteristic		Participants	
**Region of origin, n (%)**			
	Campania		83 (33.2)
	Veneto		167 (66.2)
Sex – male, n (%)		134 (53.6)	
Age (years), mean (SD)		39.7 (13.9)	
**Age groups (years), n (%)**			
	<35		104 (41.6)
	35-49		82 (32.8)
	≥50		64 (25.6)
Time from diagnosis (years), mean (SD)		10.7 (10.6)	
**Time from diagnosis by age group (years), mean (SD)**			
	<5		87 (34.8)
	5-9		46 (18.4)
	≥10		117 (46.8)
**Diseases, n (%)**			
	Ulcerative colitis		100 (40)
	Crohn disease		142 (56.8)
	Unclassified disease		8 (3.2)
**Ongoing Therapy, n (%)**			
	Mesalazine or no therapy		112 (44.8)
	Steroids, azathioprine, or biologics		138 (55.2)
**Chronic disease^a^** **, n (%)**			
	No		214 (85.6)
	Yes		36 (14.4)

**^a^**At least one other chronic disease, including diabetes, high blood pressure, and dyslipidemia.

Table 2 and 3 shows the web survey answers of the study population (N=250). Most patients were worried about COVID-19 (for Question 1, enough: n=77, 30.8%; much/very much: n=140, 56.0%) because of social distancing or going to crowded places, such as supermarkets and food shops (for Question 3, enough: n=32, 17.2%; much/very much: n=164, 65.6%). Many patients also felt disturbed and tense when thinking of SARS-CoV-2 infection (for Question 5, enough: n=77, 30.8%; much/very much: n=115, 46.0%). Moreover, patients with IBD felt more vulnerable to COVID-19 (for Question 2, enough: n=70, 28.0%; much/very much: n=109, 43.6%) and depressed (for Question 7, much/very much: n=22, 88.8%) due to their condition. Most patients (148/250, 59.2%) did not want to go to outpatient clinics at the hospital, and more than 70% (187/250) of patients preferred to undergo remote medical examination.

**Figure 2:**
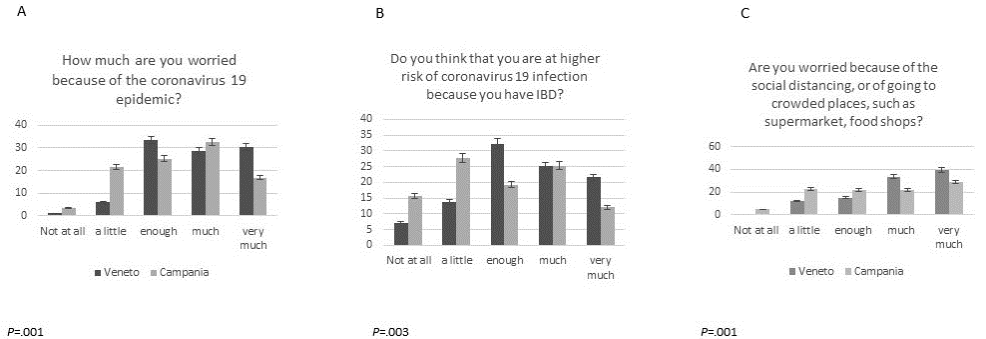
Comparison of COVID-19 survey results between participants from different regions of origin (Veneto vs Campania) for Questions 1-3 (A-C).

**Figure 3:**
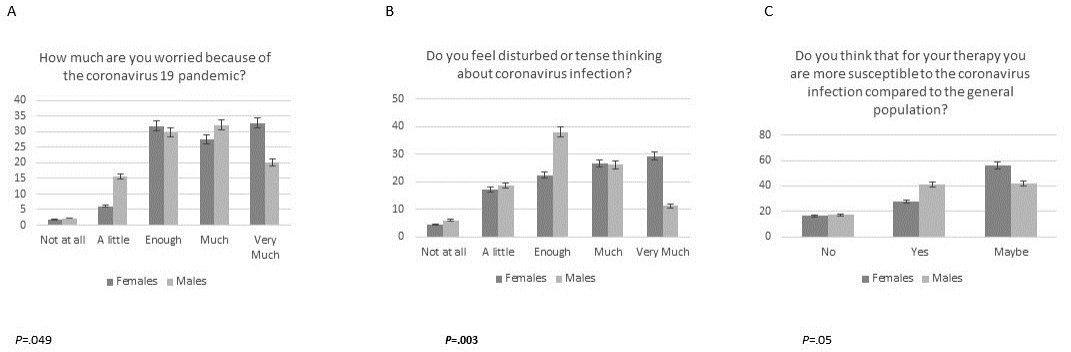
Comparison of COVID-19 survey results between male and female participants for Questions 1, 5, and 10 (A-C).

We tested for differences in survey answers between subgroups. Based on patients’ answers to Question 7 (“I feel depressed because of my disease”), younger patients (<35 years) felt significantly more depressed (answered “much” or “very much”) than older patients (≥50 years) (age: <35 years: 96/104, 92.3%; 35-50 years: 73/82, 89.02%; ≥50 years: 53/64, 82.82%; *P*=.04). Moreover, based on patients’ answers to Question 9 (“Are you happy with telemedicine remote visits?”), younger patients preferred remote medical control (answered “yes, perfect”) significantly more than the older patients (age: <35 years: 80/104, 76.9%; 35-50 years: 64/82, 78.05%; ≥50 years: 43/64, 67.2%; *P*=.04). Figure 2 shows that patients from the red zone of Veneto were more worried about SARS-CoV-2 infection (Question 1: *P*=.001), felt that they were at a higher risk of infection due to IBD (Question 2: *P*=.003), were more afraid of going to crowded places (Question 3: *P*=.001), were more tense and disturbed thinking about COVID-19 (Question 5: *P*=.001), and were more depressed due to their condition (Question 7: *P*=.007) compared to patients from Campania. Women were more worried (Question 1: *P*=.049) and felt more disturbed and tense thinking about COVID-19 (Question 5: *P*=.003) than men. However, men felt more susceptible to the virus due to their therapy (Question 10: *P*=.05) (Figure 3). More immunosuppressed patients thought that infection might interfere with their therapy (for Question 6, much/very much: 73/138, 52.9% vs 36/112, 32.1% *P*=.002) and felt more susceptible to the infection due to their therapy (for Question 10, yes: 77/138, 55.8% vs 44/112, 39.3%), *P*=.019) than patients who were not immunosuppressed. We did not find any significant differences in survey answers regarding the presence of other chronic diseases and the time from diagnosis to the point of the survey. Only one-third (83/250) of the study population received the influenza vaccination in the previous 5 months, and 47.2% (118/250) of patients reported that they would receive a vaccination against COVID-19 if available. However, a similar percentage (117/250, 46.8%) of patients had doubts about COVID-19 vaccination.

**Table 2:** Results of the survey administered to patients with inflammatory bowel disease for evaluating COVID-19 perception (Questions 1-9).

Question	Not at all (%)	A little (%)	Enough (%)	Much (%)	Very much (%)
1. How much are you worried because of the coronavirus 19 pandemic?	2	11.2	30.8	30	26
2. Do you think that you are at higher risk of coronavirus 19 infection because you have IBD?	10	18.4	28	25.2	18.4
3. Are you worried because of social distancing or of going to crowded places, such as supermarkets, food shops?	1.6	15.6	17.2	29.6	36
4. Do you think that the coronavirus 19 information is excessive?	40.4	23.6	20.8	9.6	5.6
5. Do you feel disturbed or tense thinking about coronavirus infection?	5.2	18	30.8	26.4	19.6
6. Are you worried that the coronavirus infection may interfere with your own therapy?	17.2	15.6	23.6	19.6	24
7. I feel depressed because of my disease	0.8	3.6	6.8	34	54.8
8. I am threatened by having the disease	1.6	5.6	16	47.6	29.2
9. Are you happy with telemedicine remote visits?	14 (I would like to speak with the doctors)	5.2 (No, I do not feel like being in care)	74.8 (Yes, perfect)	6 (I’m afraid I can’t answer everything)	0

**Table 3:** Results of the survey administered to patients with inflammatory bowel disease for evaluating COVID-19 perception (Questions 10-14).

Question	No (%)	Yes (%)	Maybe (%)
10. Do you think that for your therapy you are more susceptible to the coronavirus infection compared to the general population?	16.8	48.4	34.8
11. Are you afraid that pandemic reduces your care by physicians to less than it would be necessary?	48	29.2	22.8
12. Are you reluctant to go to hospital, because of coronavirus infection?	28.8	59.2	12
13. Did you undergo seasonal flu vaccination?	67.6	32.4	0
14. Would you like to undergo a vaccination for coronavirus, when it becomes available?	6	47.2	46.8

## Discussion

This cross-sectional study reports on patients with IBD from two Italian regions and their perception of the COVID-19 pandemic during the quarantine. We found that patients with IBD were particularly worried, as they felt more vulnerable to SARS-CoV-2 infection due to their chronic disease and their immunosuppressant therapy. Patients with IBD have reported a low quality of life, disability, and poor sleep quality [17-19]. Thus, the COVID-19 pandemic has worsened an already precarious condition. Further, the unfiltered amount of information from social media during the days of the epidemic has confused and scared the Italian population [20].

Patients with IBD are likely to feel that they are at a higher risk of infection compared to the general population [5]. Our data indicated that patients with IBD from the Veneto region were more worried about SARS-CoV-2 infection than patients from Campania. Veneto was one of the first Italian regions where SARS-CoV-2 infection was initially observed, and it remains one of the regions with a high number of COVID-19–related deaths (Figure 1) [21]. Therefore, the high local spread of COVID-19 has likely affected the perception of patients.

Our survey showed similar results to those of another study, which indicated that a 30-day isolation for trained personnel, such as astronauts, do not have a significant impact on brain activity, neurotrophic factors, cognition, and mood, even though the stress levels of trained personnel significantly increased during isolation [22]. However, it is conceivable that behavioral and organic consequences may follow social isolation due to COVID-19 social distancing measures [23,24]. For example, similarly to other coronaviruses, changes in endocrine and immune response may occur, as millions of people worldwide are isolated in quarantine for minimizing the transmission of SARS-CoV-2, and social isolation can lead to neuroendocrine-immune changes [25].

According to the European Crohn's and Colitis Organisation, patients with IBD should have followed the national recommendations. However, some extra caution was needed. The recommendations published during the beginning of the pandemic suggested postponing the start of treatment with immunosuppressive drugs and biologics whenever possible [26,27]. When this was not possible, screening for SARS-CoV-2 infection should have been performed before starting biologics [28,29]. Moreover, to reduce access to hospitals and transport, the postponement of any elective or routine follow-up was recommended, along with the alternative to perform remote medical examinations [12,30]. Our study showed that most patients would be happy with remote medical examinations. However, older patients were not as happy with this alternative.

Our survey showed that men felt more susceptible to SARS-CoV-2 infection than women, but women felt more worried and tense about infection. This is likely due to both the country's National Health Institute and the media simultaneously reporting that men represent nearly 60% of people who tested positive for the SARS-CoV-2 virus and more than 70% of those who were infected with SARS-CoV-2 have died.

About two-thirds of our sample reported they had not received the influenza vaccination. This is relevant because the current guidelines recommend that patients with IBD should receive an influenza vaccination every year and pneumococcal vaccination with a booster every 5 years [31]. However, our percentage of patients who were immunized for influenza was similar to those in other studies [32]. Some patients also mentioned their fear of flu vaccination and vaccinations in general. This is likely in line with the high percentage of subjects that answered “maybe” to Question 14, which asks about receiving a future SARS-CoV-2 vaccination.

Our survey also shows that patients with IBD perceived immunosuppressive and immunomodulatory therapy as an added risk to general infection. Some studies have supported this concept, reporting a higher risk of severe and opportunistic infections in patients with IBD compared to control patients [6,7]. However, other data have not suggested an increased risk in infection [9]. Patients with IBD often ask their doctors about their risk of infection, how they should behave, and what measures they should take. A multicenter study conducted in Italy showed that active IBD, old age, and comorbidities were associated with poor COVID-19 outcomes, whereas IBD treatments were not [33].

Our study had several strengths. First, to the best of knowledge, our study is the first to report on patients with IBD and their perception of the COVID-19 pandemic. Second, our participants came from two large Italian cohorts of patients with IBD who lived in similar epidemic settings. Additionally, more than half of the eligible patients responded to our survey. Thus, we achieved a larger study population than that of a recent study [34]. Lastly, we conducted this study as soon as the lockdown occurred, and at the time, it was uncertain whether COVID-19 restrictions would be lifted.

Our study also had some limitations. First, half of the patients who received the survey did not answer it, and we cannot rule out the possibility that those who responded were those most worried about the COVID-19 pandemic. Typically, optional online surveys are more likely to be answered by educated and healthy subjects [35]. Therefore, selection bias may be present in our study. Second, we did not have clinical data on IBD activity at the time of the study. As such, we could not assess the relationship between IBD activity and COVID-19 perception. Lastly, we used a standardized ad hoc questionnaire because there was no other available questionnaire in the literature. However, we formulated open questions and multiple-choice answers to avoid influencing the responses of our patients.

Our findings may be useful for future epidemics and may help to establish new ways of managing patients who live outside of hospital areas or are unable to access tertiary outpatient clinics for routine care due to their clinical conditions.

In conclusion, the results of our survey demonstrate that the lockdown had a significant impact on the psychological aspects of our patients and suggest the need for increasing communication with patients with IBD to ensure they receive adequate health care, correct information, and proper psychological support. Our results also indicate that remote medical visits may be well received by young patients with IBD and that IBD centers should implement a remote health care approach. Finally, our survey results highlight the need for encouraging flu vaccinations, as well as future vaccinations for preventing SARS-CoV-2 infection, to the public.

## Abbreviations

IBD: inflammatory bowel disease
